# Nitrogen Fertilization Increases Root Growth and Coordinates the Root–Shoot Relationship in Cotton

**DOI:** 10.3389/fpls.2020.00880

**Published:** 2020-06-23

**Authors:** Jing Chen, Liantao Liu, Zhanbiao Wang, Yongjiang Zhang, Hongchun Sun, Shijia Song, Zhiying Bai, Zhanyuan Lu, Cundong Li

**Affiliations:** ^1^State Key Laboratory of Cotton Biology (Hebei Base)/Laboratory of Crop Growth Regulation, College of Agronomy, Agricultural University of Hebei, Baoding, China; ^2^Institute of Cotton Research of Chinese Academy of Agricultural Sciences, Anyang, China; ^3^Hebei Academy of Agriculture and Forestry Sciences, Shijiazhuang, China

**Keywords:** cotton, N fertilization, root morphology, root distribution, shoot biomass

## Abstract

The root system plays an important role in the growth and development of cotton, and root growth is closely related to shoot growth, both of which are affected by N availability in the soil. However, it is unknown how N affects root growth and the root–shoot relationship under various N rates in the Yellow River Basin, China. Thus, the aim of this study was to assess the impacts of the application rate of N on root growth and the root–shoot relationship, to provide insight into the N regulation of root and shoot growth and N efficiency from the perspective of the root system. A field experiment conducted in 2014 and 2015 was used to determine the effects of N rates (0, 120, 240, and 480 kg ha^–1^) on root morphology, root distribution, the root–shoot relationship, and cotton yield. A moderate N fertilization rate (240 kg ha^–1^) increased root length, root surface area, and root biomass in most soil layers and significantly increased total root growth and total root biomass by more than 36.06% compared to the 0 kg ha^–1^ treatment. In addition, roots in the surface soil layers were more strongly affected by N fertilization than roots distributed in the deeper soil layers. Total root length, total root surface area, and root biomass in the 0–15 cm layer were significantly correlated with shoot biomass and boll biomass. In the 60–75 cm layer, total root length, total root surface area, and root length were significantly positively correlated with seed cotton yield. The application of a moderate level of N markedly increased total shoot biomass, boll biomass, and seed cotton yield. Our results show that increased shoot and boll biomasses were correlated with a significant increase in the root system especially the shallow roots in the moderate N treatment (240 kg ha^–1^), leading to an increase in cotton seed yield.

## Introduction

The root system is the main organ for nutrient absorption in plants, and it can synthesize and transport physiological activators (Tian et al., 2009; Mohd et al., 2013). Roots are therefore of great importance for crop growth, and their growth affects shoot growth and crop yield (Vamerali et al., 2003; Zhang et al., 2009; Wang et al., 2014; Ota et al., 2020) by acting as the “receptors” in a crop plant’s perception of environmental changes. Roots also regulate plant growth and development (King et al., 2003; Fahong et al., 2004). Arabidopsis modulates the efficiency of root N acquisition efficiency in response to the N demands of shoots. Shoot-to-root mobile CEPD-like 2 evalutaes shoot N status to systemically regulate nitrate uptake in Arabidopsis (Ota et al., 2020). Moderate N treatment (240 kg ha^–1^) coordinate root and shoot development. Grain yield and N use efficiency are positively correlated with the active absorption area, and negatively correlated with the root-to-shoot ratio, after the mid-tillering stage in rice (Xu et al., 2018). N deficiency is more likely to promote water absorption and N accumulation at the same root surface area level, which has been shown to lead to a higher dry mass in maize seedlings (Niu et al., 2020). N plays an important role in root and shoot communications and is critical for maximizing plant productivity and agronomic applications (Gu et al., 2018). Roots senses internal and external N changes, and coordinate developmental processes accordingly. In recent years, the root system has attracted increasing attention (Lynch, 2007; Ren et al., 2018), with studies focusing on the factors which affect root growth and distribution (Nacry et al., 2013) and the changes in the root-shoot system due to irrigation, fertilization, and straw management (Luo et al., 2014; Pu et al., 2016; Chen Z. K. et al., 2018), among which N fertilization has been regarded as the key factor in root system development.

N is a key factor in cotton production and high cotton yield can be acquired via the application of optimal fertilizer rates, which can present practical difficulties (Bondada and Oosterhuis, 2001; Ali et al., 2007; Rochester et al., 2009; Polychronaki et al., 2012; Devkota et al., 2013; Klikocka et al., 2016; Li et al., 2017). Insufficient N fertilizer application causes premature senescence, while excessive application causes late ripening and increases environmental pollution. Recent studies have measured the impacts of N fertilization on cotton (Pettigrew and Adamczyk, 2006; Alitabar et al., 2012; Zhang et al., 2012; Muharam et al., 2014; Luo et al., 2018). Root growth is significantly affected by N fertilization, for example, Gaudin and Tian showed that low N levels enhanced root elongation (Tian et al., 2008; Gaudin et al., 2011). In contrast, Fan et al. (2010) showed that increased N application increased root length and root biomass. While Wang et al. (2005) noted that high N availability reduces root biomass. Root distribution is also influenced by N levels. Zhang et al. (2017) suggested that N can affect the distribution of roots in the soil. The optimal application rate of N fertilizer might increase the distribution of roots in the layer in which the fertilizer was applied, promoting nutrient absorption, and increased the photosynthetic capacity of cotton plants. Comfort et al. (1988) suggested that excessive N application can inhibit the growth of deep roots. Root growth will affect the above ground growth of the plant. Asif et al. (2020) showed that the morphological characteristics of the root system may be an important feature for improving N use efficiency in cotton. Luo et al. (2015) demonstrated that cotton root activity at a depth of 40–120 cm was significantly correlated with photosynthetic rate and was significantly affected by N levels. The impact of N on the vertical distribution of cotton roots is an important factor influencing the photosynthetic ability of leaves (Luo et al., 2015). These observations showed that shoot growth and yield formation are affected by root growth, which could therefore reflect the status of the cotton plant’s growth. The flowering and boll period is a critical, N-sensitive period of cotton growth (Bange et al., 2004). Previous studies have mostly focused on the root growth of cotton in the Northwest Inland region of China, in the Southeastern region of the United States (Sainju et al., 2005) and in Australia (Hulugalle et al., 2014). The climatic conditions and planting patterns of the Yellow River Basin Region of China are different from these regions. In this region, cotton is generally planted at a density of 45,000 ∼ 60,000 plants per hectare, and is only twice irrigation during the whole growth period, once before sowing, and again during the flowing and boll period. Cotton plant heights reach110∼120 cm under this pattern, and this aboveground growth is different from to that seen in the cotton growing regions of northwest China, in the United States and in Australia. Previous studies were conducted in either polyvinyl chloride tubes (Luo et al., 2015; Chen Z. K. et al., 2018), greenhouses (Dong et al., 2008; Asif et al., 2020), or in a wheat-cotton intercropping system. It is unknown how N affects root growth and root distribution and how roots affect shoot biomass accumulation under various N application rates in field conduction under broad irrigation in the Yellow River Basin in China.

We therefore proposed the following hypothesis: In the Yellow River Basin, N influence on root growth and distribution of cotton in different soil layers will affect the shoot growth and seed cotton yield. Nitrogen’s influence on root growth will affect the shoot, and in this experiment, the roots in which soil depth mostly influence the shoot will also be further examined. To test this hypothesis, we conducted a field experiment in which the N supply was altered to investigate the root morphology, distribution in the soil, and root–shoot relationship during cotton’s flowering and boll periods in the Yellow River Basin of China. The aims of the study were to clarify how the amount of N impacts cotton root and shoot formation, evaluate the relationship between the root and shoot systems, and to understand at which soil depths roots were more responsive to N treatment and effective at promoting shoot biomass. This study should provide insights into how to achieve an effective root system and thus cultivate a productive root–shoot system.

## Materials and Methods

### Experimental Design

The field study was carried out in 2014–2015 at the agricultural station in Baoding (38.85°N, 115.30°E), Hebei Agriculture University, the Yellow River Valley, Hebei, China. The soil texture was loam, and the total N (1.13 g kg^–1^), available N (33.66 mg kg^–1^), organic matter (16.4 g kg^–1^), available P (15.36 mg kg^–1^), and available K (191 mg kg^–1^) were measured. NaHCO_3_ (0.5 mol L^–1^) and NH_4_OAc (1 mol L^–1^) were used as extractants to determine available P and K, respectively, (Bao, 2000). The soil pH was 8.17. The soil fertility level was intermediate. The experiment was repeated in the same plots over 2 years (i.e., the 2014 and 2015 growing seasons).

Jimian 958,a transgenic Bt cotton cultivar, was used in this study to test its performance tested under four different N (in the form of urea) fertilization treatments. The four treatments were as follows: control (0 kg ha^–1^N, N0), low N (120 kg ha^–1^N, N1), moderate N (240 kg ha^–1^N, N2), and high N (480 kg ha^–1^N, N3). The fertilizer treatments are detailed in Table 1. Half of the N was applied during the plowing before sowing, with the rest applied manually in bands near the row at the flowering stage and the peak boll-setting stage, at a depth of 5 cm. Each plot received 135 kg ha^–1^ P_2_O_5_ and 75 kg ha^–1^ K_2_O as basal fertilizer.

**TABLE 1:** Fertilizer application information.

Treatment	Fertilizer amount (kg ha^–^^1^)			Basal N fertilizer (kg ha^–1^)	Flowering stage fertilizer (kg ha^–1^)	Peak boll-setting stage fertilizer (kg ha^–1^)
	N	P_2_O_5_	K_2_O	N	N	N
N0	0	135	75	0	0	0
N1	120	135	75	60	36	24
N2	240	135	75	120	72	48
N3	480	135	75	240	144	96

*N fertilizer: urea, N 46%; phosphate fertilizer: calcium superphosphate, P_2_O_5_, 12%; potassium fertilizer: potassium chloride, K_2_O, 60%.*

The study was set up as a completely randomized design, three replicates per treatment, and the whole experiment repeated twice. The area of each replicate was 115 m^2^ (with a length and width of 11 and 10.5 m, respectively), and each plot contained fourteen rows. Cotton was planted on the 24 April 2014 and 22 April 2015 using the mechanical sowing method with interrow spacings of 0.50 and 1.00 m, with approximately four seeds dropped into each hill. At the two-leaf stage, plants were thinned to a density of 45,000 plants ha^–1^.

Plastic film mulch covered the seeds after sowing (a common practice in conventional high-yield cultivation in China). At the point of seedling emergence, the film was cut to free seedlings from the mulch. Vegetative branches were removed at the squaring stage (on 11 June 2014 and on 14 June 2015) and the apical meristem of the main stem was removed according to local practices (on 20 July in 2014 and on 21 July in 2015). In late June of each year, plots were irrigated using the flooding method. Other cultivation management practices, including hoeing and weed and pest control, were performed as per conventional high-yield cultivation practices as needed.

### Sampling

Root samples were excavated during the flowering and boll period (14 and 19 August 2014 and 2015, respectively), using a root canal drill with an inner diameter of 70 mm. Three cotton plants were randomly selected from each treatment and samples for each plant were collected at each of three locations 10 cm (horizontal distance) from the cotton plant. Sampling was conducted at eight depths at each location (0–15, 15–30, 30–45, 45–60, 60–75, 75–90, 90–120, and 120–150 cm). All sample soil cores in each soil depth and location were placed in a separate 0.15 mm mesh bag. The outside of each mesh bag was rinsed with water to remove soil particles until only the roots and some large debris remained. These were then brought into the laboratory. Roots were collected from the samples with tweezers, and initially put in a transparent box with some water. Roots were then placed separately in a transparent polyvinyl chloride (PVC) box containing 2 mm water. The roots were scanned using an EPSON-V700 scanner with WinRHIZO 7.6.1 software to measure root length, root surface area, root projected area, and root volume. Roots were then oven-dried and the biomass was weighed with an electronic balance (four-digit accuracy). The nine biological replicates from the same soil depth were used in variance analyses for each treatment.

Three plants in the central rows were concurrently sampled to measure shoot biomass characteristics, including total shoot, leaf, stem, and boll biomasses. The weights were recorded after they had been dried in an oven at 105°C for 30 min and 80°C for 6 h. Three replicates per sampling.

To obtain the seed cotton yield, plants in the middle rows of each plot were harvested twice (on 9 October and 8 November 2014 and on 6 October and 5 November 2015). After the cotton seeds were air dried, seed cotton yield was measured using an electronic balance.

### Climatic Data

**FIGURE 1:**
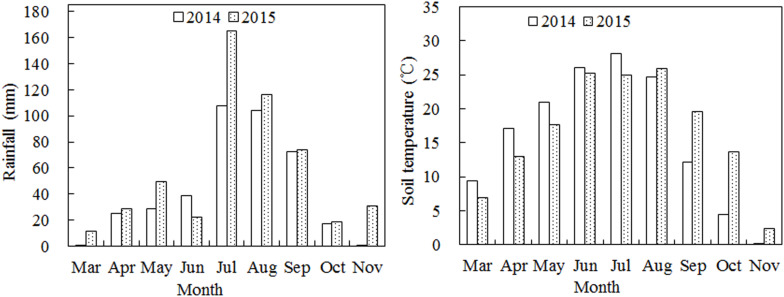
Monthly rainfall and mean temperature during the growth stages of cotton in 2014 and 2015.

Weather conditions were measured using a weather station near our experimental field. The total rainfall during the growth period in 2014 and 2015 was 396.0 and 517.9 mm, respectively, (Figure 1). The rainfall was mainly concentrated in July, August, and September, with the total rainfall during July to September being 284.2 and 355.6 mm in 2014 and 2015, respectively, accounting for 71.8 and 68.7% of the total annual rainfall. The average soil temperature varied widely during the growth period with the maximum soil temperature was observed in June, July, and August at a depth of 0–20 cm.

### Statistical Analysis

A one-way analysis of variance (ANOVA) was conducted using SPSS version 17.0. The ANOVA was conducted using Duncan’s test and differences between treatments were considered significant at *P* < 0.05. Pearson correlation coefficients were calculated with the correlation procedure in SPSS version 17.0. The data were analyzed by year because the effects of N fertilizer and soil depth on cotton root morphology, root dry weight, root distribution, and aboveground biomass varied. There was no interaction effect of N fertilizer and year on yield; therefore, yield was analyzed using the average values across the 2-year study period.

## Results

### Impact of N Application Amount on Root Length in Various Soil Layers

**FIGURE 2:**
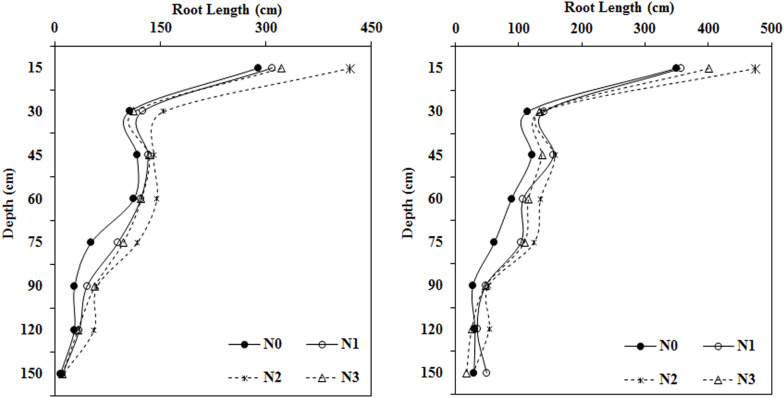
Impact of N application on cotton root length in various soil layers during the flowering and boll period (**left:** 2014; **right:** 2015).

N fertilizer application significantly promoted root length elongation at most soil depths (0–120 cm) compared with the N0 treatment (Figure 2). The application of a moderate amount of N fertilizer treatment (N2, 240 kg ha^–1^) resulted in the longest roots in most soil layers (0–120 cm).

At a soil depth of 0–15 cm, as the amount of N fertilizer applied increased, root length first increased, then decreased. Roots collected from the moderate N treatment (N2, 240 kg ha^–1^) were 44.55, 35.41, and 30.01% longer in 2014 and 35.70, 33.08, and 18.22% longer in 2015 than roots collected from the N0, N1, and N3 treatments, respectively. Roots in the moderate N treatment were longer than those collected from the N0 treatment in both years in soil collected from depths of 15–30, 30–45, and 45–60 cm. Roots in the N fertilization treatments were much longer than roots collected from the N0 treatment at depths of 60–75 and 75–90 cm. Relative to the N0 treatment, N treatments increased the root length at a depth of 60–75 cm by 74.21 to 127.10% in 2014 and by 67.58 to 101.95% during the 2015 growing season. At the 75–90 cm depth, the roots were 62.61, 112.98, and 102.16% longer in 2014 and 72.35, 88.79, and 71.82% longer in 2015 in the N1, N2, and N3 treatments, respectively, compared with the N0 treatment.

### Impact of N Application Rate on Root Surface Area in Different Soil Layers

**FIGURE 3:**
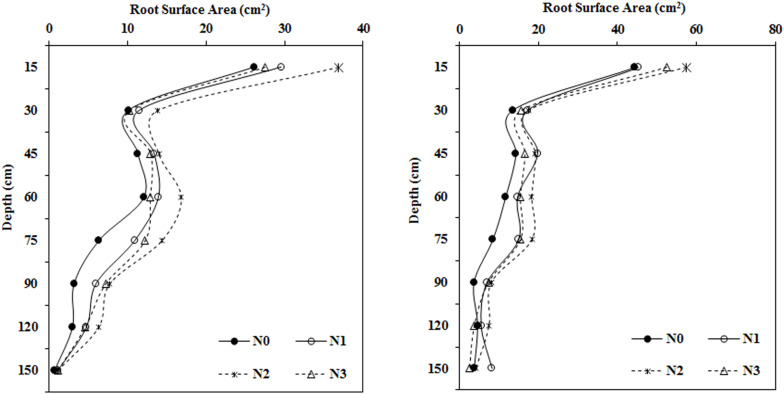
Impact of N application on cotton root surface area in various soil layers during the flowering and boll period (**left:** 2014; **right:** 2015).

In both years, the root surface area in most soil layers first increased and then decreased with the application of increasing amounts of fertilizer. This was especially apparent in the roots collected from the shallow soil layer (Figure 3). The root surface area under N2 treatment reached a maximum at depths ranging from 0–120 cm and was greatly increased compared to that in the N0 treatment. Root surface area in the moderate N treatment (N2, 240 kg ha^–1^) was significantly greater than that in the N0 treatment by 40.97, 36.85, 23.50, and 39.33% in 2014 and by 29.11, 29.58, 34.30, and 55.48% in 2015 at soil depths of 0–15, 15–30, 30–45, and 45–60 cm, respectively. N fertilization had a stronger influence on root surface area at depths of 75–90 and 90–120 cm. Overall, the root surface area under all N fertilization levels was greater than that in the N0 treatment in 2014 and 2015.

### Impact of the N Application Rate on Root Volume in Various Soil Layers

**FIGURE 4:**
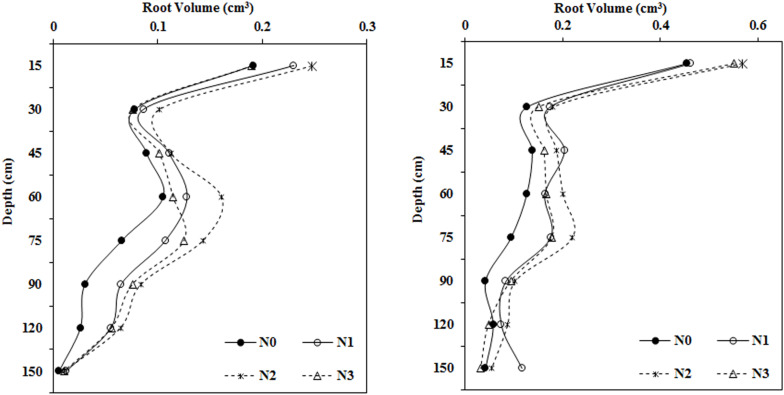
Impact of N application on cotton root volume in various soil layers (**left:** 2014; **right:** 2015).

As N fertilization increased, root volume first increased and then decreased in most soil layers, and the differences varied between treatments (Figure 4). There were no apparent differences in root volume after N treatments at soil depths of 0–15 and 30–45 cm, however, root volume in the moderate N treatment (N2, 240 kg ha^–1^) was much higher than root volume in the N0 treatment at depths of 15–30, 45–60, 60–75, 75–90, and 90–120 cm.

### Impact of N Application Rate on Root Biomass in Different Soil Layers

**FIGURE 5:**
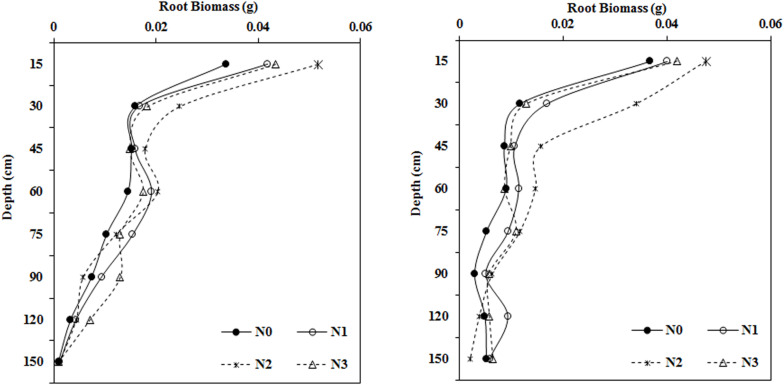
Impact of N application on cotton root biomass in various soil layers during the flowering and boll period (**left:** 2014; **right:** 2015).

Root biomass in the upper soil profile (0–60 cm) showed the same trend among the different treatments (Figure 5) with the moderate N treatment (N2, 240 kg ha^–1^) yielding the largest root biomass values in the 0–15, 15–30, and 45–60 cm soil layers in 2014 and 2015. However, different root biomass trends were observed in the deeper soil layers, for example, N increased the root biomass at a depth of 60–75 cm. The root biomass under N1 was significantly higher than that under the N0 treatment in 2014, but in 2015, root biomass was much higher in the N2 treatment than the N0 treatment. Moreover, at the 75–90 cm soil layer, root biomass reached a maximum in the N3 and N2 treatments in 2014 and 2015, respectively.

### Impact of N Application Rate on Total Root Length, Root Surface Area, Root Volume, and Root Biomass

**FIGURE 6:**
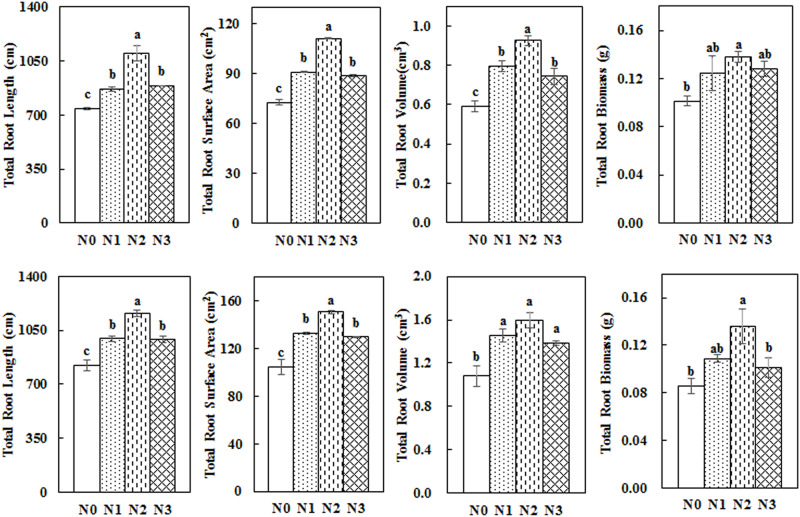
Impact of N application on the total root length, root surface area, root volume, and root biomass of cotton during the flowering and boll period (**upper:** 2014; **lower:** 2015). Different letters among treatments indicate significant differences in the index at *P* = 0.05.

Total root length, surface area, volume, and biomass first increased and then decreased with the increase in N fertilization rates (Figure 6). The values from the N2 treatment were the highest among the different treatments. N2 treatment significantly increased total root length, surface area, volume, and dry biomass by 48.80, 52.57, 56.73, and 36.06% in 2014 and by 40.97, 43.91, 47.74 and 58.39% in 2015, respectively, compared with N0.

### Impact of N Application Rates on Root Distribution in Various Soil Layers

The cotton root system was distributed primarily in the 0–15 cm soil layer, at which the main root indexes, including root length, surface area, volume, and biomass, all had the largest percentages, and accounted for more than 35.61, 32.37, 25.51, and 33.26%, respectively, of their corresponding totals (Table 2). The root system decreased with increasing soil depths and the differences between soil depths were significant in both years. Root biomass in the 0–15, 0–30, 0–45, and 0–60 cm soil depths accounted for more than 33.3, 46.9, 59.7, and 73.4% of the total root biomass in 2014 and 35.9, 52.3, 62.1, and 71.1% of the total root biomass in 2015, respectively.

**TABLE 2:** Impacts of N application on root distribution in various soil layers.

Year	Index	Root length (%)				Root surface area (%)				Root volume (%)				Root biomass (%)			
	Soil depth	N0	N1	N2	N3	N0	N1	N2	N3	N0	N1	N2	N3	N0	N1	N2	N3
2014	0–15 cm	39.18 a	35.61 a	37.84 a	36.23 a	35.8 a	32.37 a	33.04 a	33.00 a	32.35 a	28.78 ab	26.79 ab	25.51 b	33.26 b	33.56 b	37.47 a	33.84 b
	15–30 cm	14.36 a	14.33 a	13.95 a	12.65 a	13.84 a	12.59 ab	12.43 ab	11.54 b	13.14 a	10.88 ab	10.99 ab	10.27 b	15.74 ab	13.38 b	17.73 a	14.14 ab
	30–45 cm	15.79 a	15.23 a	12.76 b	15.11 a	15.53 a	14.72 ab	12.58 b	14.62 ab	15.01 a	13.97 a	12.14 a	13.74 a	15.08 a	12.91 a	12.90 a	11.73 a
	45–60 cm	15.17 a	14.05 ab	13.16 b	13.83 ab	16.60 a	15.29 a	15.15 a	14.56 a	17.83 a	16.21 a	17.44 a	14.79 a	14.35 a	15.54 a	14.79 a	13.67 a
	60–75 cm	6.94 b	10.3 a	10.69 a	10.93 a	8.77 b	12.00 ab	12.98 ab	13.76 a	11.28 a	13.57 a	15.51 a	16.76 a	10.21 ab	12.54 a	8.83 b	10.05 ab
	75–90 cm	3.79 c	5.24 b	5.44 ab	6.37 a	4.37 b	6.57 a	7.00 a	8.11 a	5.08 b	8.21 ab	9.07 a	10.14 a	7.25 ab	7.38 ab	4.21 b	10.18 a
	90–120 cm	3.71 b	4.00 ab	5.05 a	3.68 b	4.11 a	5.22 a	5.74 a	5.15 a	4.40 a	6.91 a	6.93 a	7.33 a	3.24 a	3.68 a	3.24 a	5.63 a
	120–150 cm	1.06 a	1.25 a	1.11 a	1.22 a	0.97 a	1.24 a	1.08 a	1.26 a	0.91 a	1.47 a	1.12 a	1.45 a	0.87 a	1.02 a	0.83 a	0.75 a
2015	0–15 cm	42.45 a	35.78 b	40.85 a	40.57 a	42.46 a	34.09 b	37.98 ab	40.49 a	42.02 a	31.93 b	35.19 ab	39.91 ab	43.40 a	36.91 a	35.88 a	41.07 a
	15–30 cm	13.84 ab	14.13 a	11.68 b	13.57 ab	12.83 a	12.97 a	11.56 a	12.12 a	11.74 ab	11.94 ab	11.26 a	10.86 b	14.87 a	15.34 a	24.15 a	11.59 a
	30–45 cm	14.76 a	15.64 a	13.54 a	13.84 a	13.76 a	14.90 a	12.79 a	12.82 a	12.76 a	13.99 a	11.83 a	11.69 a	10.06 a	9.82 a	11.47 a	9.94 a
	45–60 cm	10.75 a	10.79 a	11.64 a	11.72 a	11.17 a	11.09 a	12.20 a	11.93 a	11.55 a	11.25 a	12.56 a	12.04 a	10.25 a	10.51 a	11.07 a	8.47 a
	60–75 cm	7.44 b	10.39 a	10.77 a	11.03 a	8.06 b	11.26 a	12.23 a	11.96 a	8.65 b	12.05 a	13.78 a	12.89 a	6.21 a	8.76 a	8.59 a	10.58 a
	75–90 cm	3.39 b	4.82 a	4.52 a	4.84 a	3.62 b	5.28 a	5.39 a	5.72 a	4.00 b	5.79 a	6.42 a	6.77 a	3.61 a	4.71 a	4.83 a	5.76 a
	90–120 cm	3.76 b	3.48 b	4.60 a	2.62 c	4.44 a	4.17 a	5.02 a	2.95 b	5.37 a	5.12 a	5.50 a	3.49 a	5.73 ab	8.49 a	2.56 b	5.87 ab
	120–150 cm	3.60 b	4.97 a	2.41 c	1.81 c	3.66 b	6.24 a	2.83 b	2.01 b	3.91 b	7.95 a	3.46 b	2.35 b	5.87 a	5.46 a	1.44 a	6.72 a

*For each parameter, each soil layer and each index were analyzed separately. Different letters among treatments in one column indicate significant differences in the index at *P* = 0.05.*

The amount of N fertilizer affected the root distribution in various soil layers. The N2 treatment showed decreased root lengths in the upper soil layers (0–15, 15–30, and 30–45 cm) but increased the root lengths in the deeper layers (60–75, 75–90, and 90–120 cm). The differences in the root lengths between treatments at soil depths of 30–45, 45–60, 60–75, 75–90, and 90–120 cm in 2014 and 60–75, 75–90, and 90–120 cm in 2015 were significant. The distribution of root surface area and root volume largely exhibited the same trends as root length, but the differences in most soil layers were not significant in 2014; the distribution of root surface area and root volume were only significantly different in the deeper soil layers in 2015. Differences between treatments in root biomass distribution were not significant in most soil layers and differed between years. In 2014 but not 2015, N2 treatment increased root biomass distribution in 0–15 cm soil layer.

### Impact of N Application Rate on Shoot Biomass

**FIGURE 7:**
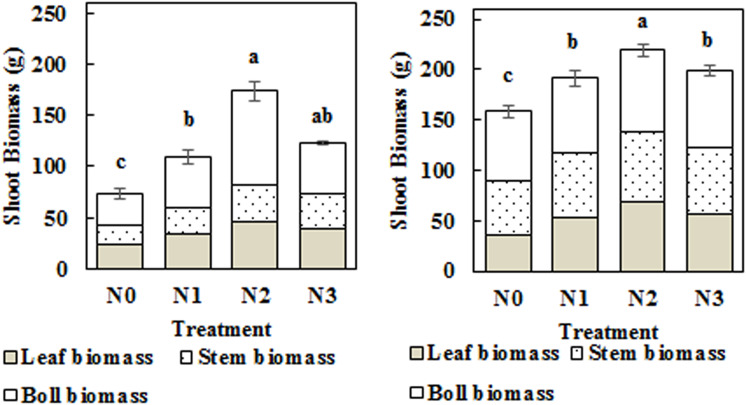
Impact of N application on cotton leaf biomass, stem biomass, and boll biomass at the flowering and boll period (**left**: 2014; **right:** 2015). Different letters among treatments indicate significant differences in the index at *P* = 0.05.

Total shoot biomass was significantly increased by N fertilization in 2014 and 2015 (Figure 7). The N1, N2, and N3 treatments significantly increased shoot biomass by 48.14, 136.37, and 67.38% in 2014 and by 20.51, 38.32, and 25.44% in 2015, respectively, compared to the N0 treatment. Boll biomass exhibited the same trend as total shoot biomass, which first increased and then decreased with the increasing application rate of N, reaching its highest values in the N2 treatment (N2, 240 kg ha^–1^). Leaf biomass and stem biomass exhibited similar trends, with the highest values observed in the N2 treatment. Furthermore, the boll biomass proportion in the N2 treatment increased to 52.66 and 37.14% in 2014 and 2015, respectively.

### Correlations Between Root Morphology Indexes in Various Soil Layers and Shoot Biomass

During the flowering and boll period, shoot biomass was correlated with root biomass (Table 3). Boll and total shoot biomasses were significantly associated with root biomass at a soil depth of 0–15 cm. Stem biomass was also significantly correlated with total root biomass. Root biomass affected shoot biomass accumulation and had the greatest impact at a soil depth of 0–15 cm.

**TABLE 3:** Correlation coefficients between root and shoot biomasses at the flowering and boll period.

Root biomass depth	2014				2015			
	Leaf biomass	Stem biomass	Boll biomass	Total shoot biomass	Leaf biomass	Stem biomass	Boll biomass	Total shoot biomass
0–15 cm	0.763	0.592	0.983**	0.914*	0.624	0.882	0.974**	0.889*
15–30 cm	0.585	0.424	0.964**	0.818	–0.413	–0.043	0.333	–0.193
30–45 cm	0.213	–0.013	0.767	0.504	–0.021	0.311	0.430	0.158
45–60 cm	0.581	0.399	0.809	0.722	0.489	0.782	0.861	0.667
60–75 cm	0.264	0.203	0.104	0.171	0.578	0.844	0.984**	0.747
75–90 cm	0.449	0.604	–0.221	0.133	0.829	0.912*	0.941*	0.906*
90–120 cm	0.848	0.937*	0.334	0.639	0.891*	0.880	0.830	0.915*
120–150 cm	0.114	–0.091	0.459	0.269	0.943*	0.743	0.454	0.837
0–150 cm (Total)	0.908*	0.928*	0.683	0.856	0.574	0.904*	0.962**	0.746

* Significant at *P* ≤ 0.05; ** Significant at *P* ≤ 0.01.

### Correlations Between Total Root Morphology Indexes and Shoot Biomass

**FIGURE 8:**
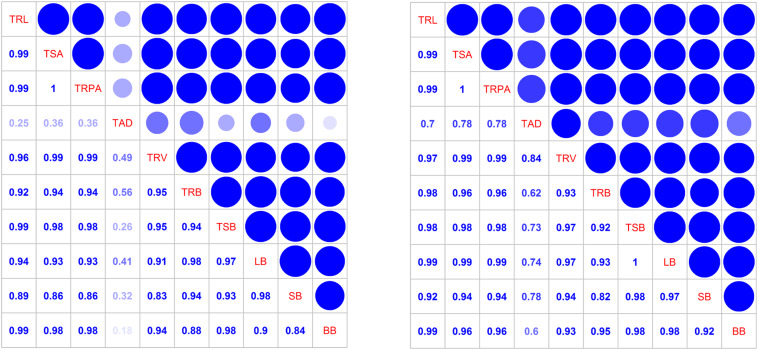
Correlation coefficients among the total root morphology indexes and shoot biomass at the flowering and boll period (left: 2014; right: 2015). TRL, total root length; TSA, total surface area; TRPA, total root projected area; TAD, total average diameter; TRV, total root volume; TRB, total root biomass; TSB, total shoot biomass; LB, leaf biomass; SB, stem biomass; BB, boll biomass.

Total root length, root surface area, root projected area, and root volume were significantly correlated (Figure 8). Total root length, surface area, and root projected area were significantly associated with total shoot and boll biomasses, indicating that root morphological traits affect each other and can significantly affected shoot biomass accumulation, especially the boll biomass.

### Impact of N Application Amount on Seed Cotton Yield

**FIGURE 9:**
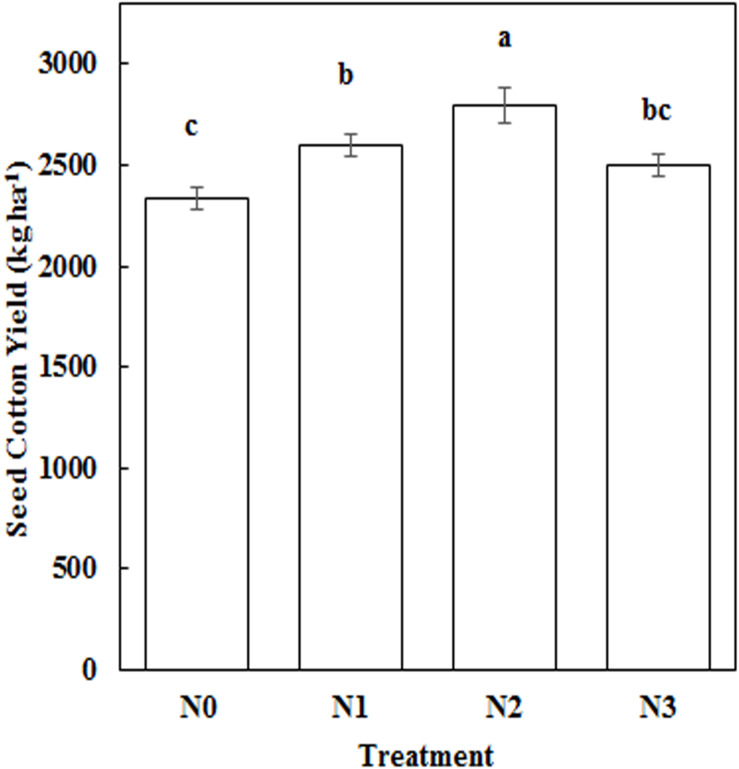
Impact of N application rate on seed cotton yield. Different letters among treatments indicate significant differences in the index at *P* = 0.05.

N fertilization increased seed cotton yields compared to N0. Seed cotton yield was 19.64% higher under N2, which is significantly different to that of N0 (Figure 9). Yield was strongly correlated with root length (30–45 cm, 60–75 cm), root surface area (60–75 cm), root volume (60–75 cm), root biomass (0–15 cm, 45–60 cm), total root length, total root projected area, total root biomass, and boll biomass in both years (Table 4). Root length (120–150 cm), root surface area (30–45 cm, 90–120 cm), root volume (90–120 cm), total root volume, and yield, all showed significant correlation with seed cotton yield in 2014. In 2015, yield was significantly associated with root biomass (15–30 cm, 30–45 cm), indicating that root morphology at the flowering and boll formation stage obviously affected seed cotton yield. The greatest impacts were observed for root length at a 60–75 cm depth, root surface area at 60–75 cm, root volume at 60–75 cm, root biomass at 0–15 cm and 45–60 cm, total root length, total root projected area, and total root biomass.

**TABLE 4:** Correlation coefficients between the different indexes and seed cotton yield under different N fertilizer treatments.

	2014	2015
Root length at 30–45 cm	0.971*	0.980*
Root length at 60–75 cm	0.977*	0.950*
Root length at 120–150 cm	0.972*	ns
Root surface area at 30–45 cm	0.980*	ns
Root surface area at 60–75 cm	0.967*	0.972*
Root surface area at 90–120 cm	0.996**	ns
Root volume at 60–75 cm	0.950*	0.963*
Root volume at 90–120 cm	0.959*	ns
Root biomass at 0–15 cm	0.984*	0.949*
Root biomass at 15–30 cm	ns	0.952*
Root biomass at 30–45 cm	ns	0.957*
Root biomass at 45–60 cm	0.972*	0.989*
Total root length	0.962*	0.963*
Total root projected area	0.987*	0.899*
Total root volume	0.996**	Ns
Total root biomass	0.973*	0.964*
Boll biomass	0.954*	0.971*

* Significant correlation with seed cotton yield at *P* ≤ 0.05; ** significant correlation with seed cotton yield at *P* ≤ 0.01; ns, no significant correlation with seed cotton yield.

## Discussion and Conclusion

This study revealed how N affects cotton root morphology and distribution, as well as how the root system affects shoot biomass and cotton seed yield. We showed that root systems were larger under moderate and high N fertilization rates than under other treatments. In addition, the root length, surface area, volume, and biomass in most soil layers were the highest under N2 treatment fertilization and were much higher than in the control. Root lengths collected in the upper soil layers were decreased in the N2 treatment fertilization treatment. Boll biomass was correlated with total root length, surface area, projected area, and biomass in the 0–15 cm layer. Seed cotton yield was strongly associated with total root length, total root projected area, total root biomass, boll biomass, and root morphology in the 60–75 cm soil layer.

Normal root morphology, which promotes a high crop yield, is the foundation of nutrient and water uptake by plants. Root length and surface area are appropriate indexes for describing a root system (Amato and Ritchie, 2002). In this study, we found that root length, surface area, volume, and biomass generally decreased with an increase in soil depth, findings which are consistent with Chen et al. (2010) and Coelho et al. (2003). Root length, surface area, volume, and biomass were highest in the 0–15 cm soil layer, similar to the findings of Min et al. (2014) study of drip irrigation with saline water. N fertilizer promoted root growth in most soil layers, which is not supported by Min et al. (2014), however, this may be due to their use of saline water irrigation, where N could have increased salt stress. The moderate N fertilization rate used in this study significantly increased root elongation and resulted in the greatest root length, surface area, and volume in most soil layers, with root length being consistent with the results of Chen J. et al. (2018) who used the minirhizotron method throughout the whole growing period at a distance of 25 cm. Zhang et al. (2017) showed that N fertilization increased root length density and root activity. We found that the highest root biomass was obtained in the N2 treatment, therefore, we suggest that root elongation was inhibited by either N deficiency or excess. N deficiency can lead to low root activity and water consumption (Zhang et al., 2017) and decrease the production of reactive oxygen species (ROS) in roots (Chen Z. K. et al., 2018), resulting in low root biomass accumulation. Excess N can increase aboveground organ growth and decrease root growth (Zhang et al., 2017).

We found that the root system was primarily distributed in the shallow soil layer, with the highest values for root length, surface area, and biomass measured in 0–15 cm layer. The root length, surface area, and biomass in the 0–30 cm soil depth accounted for approximately 50% of the corresponding totals, which agrees with the results described by Min et al. (2014), who showed that root biomass was mostly distributed in the 0–20 cm soil layer. However, that study (Min et al., 2014) found that 85–90% of total root biomass was distributed in the 0–20 cm layer, which is a much higher range than the corresponding percentages in our study. This difference may be due to different irrigation methods between the two studies as drip irrigation distributes more water in the shallow soil layers than flood irrigation. In this study, the N fertilization rate impacted the root distribution in all soil layers. Relative to the N0 treatment, the root length in the moderate N treatment (N2, 240 kg ha^–1^) was lower in surface soil and higher in deeper soil, and the differences were significant in the 60–75 cm, 75–90 cm, and 90–120 cm layers. These results indicate that the N2 treatment increased the root length distribution in deeper soil layers, which can be expected to promote photosynthesis and water potential in leaves (Luo et al., 2014). Root biomass exhibited a different tendency. Compared to N0, N2 treatment increased the distribution of root biomass in surface soil layers but decreased it in deeper soil layers. This phenomenon might have been due to roots in the deeper soil layers being mostly tender, thin roots with a high-water content (Nielsen et al., 2001), meaning root morphology only increased in response to N fertilizer in the deeper soil layers.

Total root length, surface area, volume, and dry biomass were affected by N fertilization, with the greatest values observed in the 240 kg ha^–1^N treatment. Total root length, surface area, projected area, and biomass in the 0–15 cm layer were significantly associated with total shoot and boll biomasses, indicating that root morphological traits significantly affected shoot biomass accumulation, especially the boll biomass. Seed cotton yield was strongly associated with total root length, projected area, biomass, boll biomass, root morphology (60–75 cm soil layer), and root biomass (0–15 cm and 45–60 cm soil layer), illustrating that the root morphology at the flowering and boll formation stage significantly affected seed cotton yield. This was especially evident for root morphology in the 0–15, 45–60, and 60–75 cm soil layers. This is similar to the findings of Zhang et al. (2017), who showed that shoot biomass was positively correlated with root length density and root activity in the 40–120 cm soil layer. Our study showed that with the significant increase of the root system in the N2 treatment (240 kg ha^–1^), the shoot and the boll biomasses increased proportionally, leading to an increase in the cotton seed yield. In contrast, N deficiency suppressed root and shoot biomass accumulation, most likely because a lack of N fertilization can be expected to result in a low N concentration in the soil, and a subsequent decline in N uptake (Yang et al., 2013), inhibiting root elongation, especially in the surface soil layers. N deficiency in roots could inhibit shoot growth, which in turn could restrict root growth, resulting in a decrease in yield. Although the chlorophyll content in leaves increases with excess N (Dong et al., 2012), the balance of carbon and N metabolism can be disturbed by high N fertilizer application rates (Hu et al., 2008). Therefore, under high rates of N application, the transport of photosynthetic products to reproductive organs will decrease (Zhou et al., 2011). Under these conditions, the growth of the source system in the leaf then becomes the priority, and root growth is decreased. Thus, the balance of vegetative and reproductive growths becomes disrupted due to nutrient excess (Dong et al., 2008). The physiological metabolism and N contents in seeds and bolls were increased with excessive N application, resulting in a decrease in boll weight and fiber quality (Wang et al., 2012). Our results demonstrate that the application of a moderate amount of N fertilizer facilitates root growth and the coordination of root and shoot biomass accumulation. These results may be related to the increase of physiological activity in roots, N uptake, and the activity of reaction centers of photosystem II (Shenker et al., 2003; Chen et al., 2018). Moderate available N could improve assimilate transport from source to sink, which could increase biomass in the reproductive organs (Yang et al., 2011). Future studies should be conducted to determine precisely how root function changes under various levels of N application.

The amount of N applied to a field significantly affected root morphology and distribution. The moderate treatment improved root growth in each soil layer and increased the total root length, surface area, volume, and biomass. Root morphology and biomass were most strongly affected in the upper soil layers by the application of N. These findings indicate that moderate N fertilization (N2 treatment) can promote root growth, especially for shallower roots (0–15 cm), thereby increasing the biomass of roots and shoots and achieving high seed cotton yield.

## Data Availability Statement

All datasets presented in this study are included in the article/Supplementary Material.

## Author Contributions

JC, LL, and CL conceived and designed the experiments. JC and LL performed the experiments. JC and ZW analyzed the data. JC, LL, YZ, HS, SS, ZB, and ZL contributed to the reagents, materials, and analytical tools. JC, LL, and ZW wrote the manuscript. All authors contributed to the article and approved the submitted version.

## Conflict of Interest

The authors declare that the research was conducted in the absence of any commercial or financial relationships that could be construed as a potential conflict of interest.
